# Supplementary material for: Effects of a multimodal physical therapy approach on breast cancer-related lymphedema: a retrospective pre-post study
Source: Sci Rep. 2025 Nov 21;15:41175. doi: 10.1038/s41598-025-27786-0 (PMC12638769; doi:10.1038/s41598-025-27786-0)
Supplement: Supplementary file 1 — Supplementary Material 1 [file 41598_2025_27786_MOESM1_ESM.docx]

**Effects of a multimodal physical therapy approach on breast cancer-related lymphedema: A retrospective pre-post study**

Divya Varshini ^1^, Mahalakshmi Venugopalan ^2^, Ashokan Arumugam ^3,4,5,6^, Filippo Migliorini ^7,8,9^, Nicola Maffulli ^10,11,12^, Rajkumar Kottayasamy Seenivasagam ^13^

1. SRM College of Physiotherapy, Faculty of Medicine and Health Sciences, SRM Institute of Science and Technology (SRMIST), Kattankulathur, India
2. Department of Physiotherapy, Government Hospital, Madukkarai, Coimbatore, India
3. Department of Physiotherapy, College of Health Sciences, University of Sharjah, P.O. Box: 27272, Sharjah, United Arab Emirates
4. Neuromusculoskeletal Rehabilitation Research Group, RIMHS–Research Institute of Medical and Health Sciences, University of Sharjah, P.O. Box: 27272, Sharjah, United Arab Emirates
5. Sustainable Engineering Asset Management Research Group, RISE–Research Institute of Sciences and Engineering, University of Sharjah, P.O. Box: 27272, Sharjah, United Arab Emirates
6. Department of Physiotherapy, Manipal College of Health Professions, Manipal Academy of Higher Education, Manipal, Karnataka, India
7. Department of Trauma and Reconstructive Surgery, University Hospital of Halle, Martin-Luther University Halle-Wittenberg, 06097 Halle (Saale), Germany
8. Department of Orthopaedic and Trauma Surgery, Academic Hospital of Bolzano (SABES-ASDAA), via Lorenz Böhler 5, 39100, Bolzano, Italy.
9. Department of Life Sciences, Health, and Health Professions, Link Campus University, Via del Casale di San Pio V, 00165 Rome, Italy
10. Department of Orthopaedic and Trauma Surgery, Faculty of Medicine and Psychology, University La Sapienza, 00185 Roma, Italy
11. School of Pharmacy and Bioengineering, Keele University Faculty of Medicine, Stoke on Trent ST4 7QB, UK
12. Centre for Sports and Exercise Medicine, Barts and the London School of Medicine and Dentistry, Mile End Hospital, Queen Mary University of London, London E1 4DG, UK
13. Department of Surgical Oncology, PSG Institute of Medical Sciences and Research, Coimbatore, India

**Mail authors**

Divya Varshini: [divyayadav2596@gmail.com](mailto:divyayadav2596@gmail.com)

Mahalakshmi Venugopalan: [maha77physio@gmail.com](mailto:maha77physio@gmail.com)

Ashokan Arumugam: [ashokanpt@gmail.com](mailto:ashokanpt@gmail.com)

Filippo Migliorini: [filippo.migliorini@uk-halle.de](mailto:filippo.migliorini@uk-halle.de)

Nicola Maffulli: [n.maffulli@qmul.ac.uk](mailto:n.maffulli@qmul.ac.uk)

Rajkumar Kottayasamy Seenivasagam: [drksrajkumar@gmail.com](mailto:drksrajkumar@gmail.com)

**Corresponding author**

Prof. Dr. med. Filippo Migliorini MD, PhD, MBA

Department of Trauma and Reconstructive Surgery

University Hospital of Halle

Martin-Luther University Halle-Wittenberg

Ernst-Grube-Street 40, 06097 Halle (Saale), Germany

E-mail: filippo.migliorini@uk-halle.de

ORCID: 0000-0001-7220-1221

**Abstract**

Despite the availability of numerous lymphedema treatments, integrating physical therapy for breast cancer-related lymphedema (BCRL) management has limited evidence. The objective is to evaluate the effect of a multimodal physical therapy approach in reducing upper limb (UL) lymphedema and improving shoulder range of motion (ROM) in women with BCRL. A retrospective study was conducted with 19 women diagnosed with BCRL. UL circumference and shoulder ROM were assessed before and after four weeks of therapy and showed significant improvement (p < 0.05). The mean difference between baseline and 4-week intervention was 2.11cm for UL circumference (95% CI: 1.54, 2.68; Cohen’s d = 1.35), 46.31^o^ for shoulder flexion (95% CI -57.5, -35.1; Cohen’s d = 1.68), 10^o^ for shoulder extension (95% CI -13.4, -6.59; Cohen’s d = 1.74), 47.36^o^ for shoulder abduction (95% CI -60.35, -34.38; Cohen’s d = 1.67), 18.94^o^ for shoulder internal rotation (95% CI -24.4, -13.4; Cohen’s d = 1.37) and 16.05^o^ for shoulder external rotation (95% CI -20.03, -12.07; Cohen’s d = 1.51). Women with BCRL showed significant improvements in lymphedema and shoulder mobility following four weeks of therapy. Further, robust randomised clinical trials are required to substantiate these findings in Indian women.

**Key Words**: breast cancer-related lymphedema, physical therapy, intermittent pneumatic compression, manual lymphatic drainage, compression garments

**Introduction**

According to the global burden of disease study, breast cancer was the third most prevalent cancer in 2017, with an estimated 2.0 million incident cases (95% Uncertainty Interval (UI), 1.9-2.0 million). In 2019, breast cancer was the most prevalent cause of cancer-related Disability-Adjusted Life Years (DALYs), deaths and Years of Life Lost (YLLs) among females worldwide ^(1)^. Approximately 90% of Breast Cancer Related Lymphedema (BCRL) cases occur within the first two years after radiation therapy, adjuvant chemotherapy and surgical interventions ^(2)^.

The diagnosis of lymphedema could be based on a difference of >200 ml in arm volume, >20 mm in arm circumference, or an increase of >10% in volume between the abnormal and normal arms ^(3)^. The underlying pathophysiology of this condition involves draining interstitial fluid from the skin and subcutaneous tissue by superficial lymphatic capillaries into deeper, larger lymphatic vessels, which then collect and move into axillary lymph nodes. Axillary dissection or lymphangitic infiltration disrupts this mechanism, resulting in lymphedema. The leakage of lymphatic fluid, rich in proteins, into the interstitial space causes eventual fibrosis ^(4)^. This condition poses significant challenges for breast cancer survivors because they experience multiple symptoms such as swelling, heaviness, limb weakness, reduced quality of life and decreased daily activities ^(5)^.

The International Society of Lymphology classifies limb lymphedema into four stages and is the most commonly used diagnostic system for BCRL. Stage 0 denotes subclinical or latent limb lymphedema, characterised by no evident volume gain and swelling, but an initially impaired lymphatic network. Stage I, conversely, indicates mild lymphedema, characterised by visible transient limb swelling and volume gain, which may be reduced with limb elevation. Stage II, on the other hand, indicates non-reducible lymphedema, characterised by poor pitting edema and little to no response to limb elevation, serving as an indicator of tissue changes. Finally, stage III denotes overt lymphedema, characterised by tissue changes such as fat and tissue fibrosis, hypertrophic thickened skin, and limb deformities ^(3)^.

Physical therapy plays a significant role in the treatment, prevention, and early diagnosis of secondary lymphedema ^(6)^. A previous study by Hemmati et al. ^(7)^ reported that a combination of complex decongestive therapy, such as manual Lymphatic drainage (MLD), compression therapy with a short stretch bandage, skin care, and lymphedema exercises, along with electrotherapy modalities, results in a more effective course of treatment

MLD, a specific type of manual therapy, applies delicate strokes in the direction of lymphatic movement, from the proximal to the distal segment, as part of complete decongestive therapy. MLD primarily aims to activate the functioning lymphatic veins and promote lymphokinetic activity. Before compression therapy, MLD is suggested. The treatment is not regarded as a stand-alone one ^(8)^. An air pump with inflatable auxiliary sleeves or gloves makes up an intermittent pneumatic compression (IPC) system ^(3)^. They facilitate the body’s lymphatic and venous return from distal to proximal regions ^(8)^. Similarly, a systematic review by Yao et al. found that both decongestive lymphatic therapy and intermittent pneumatic compression are effective strategies for controlling BCRL. Early implementation of compression therapy is crucial, as it represents a cornerstone in the long-term management of chronic lymphedema. Compression modalities are typically applied during the intensive phase of treatment to reduce swelling ^(9,10)^.

A systematic review revealed that regular exercise benefits BCRL management by promoting lymph angiogenesis, which may minimise radiation- and dissection-related damage. Exercise raises blood pressure and cardiac output, subsequently increasing capillary filtration and interstitial pressure, thereby facilitating the entry of fluid and proteins into lymphatic capillaries ^(11)^. Shoulder exercises activate the anterior upper arm muscles (shoulder and elbow flexors) and the posterior upper arm muscles (the deltoid and triceps brachii). Collectively, exercise promotes lymph fluid flow and drainage by increasing muscle tissue pressure while concurrently lowering venous pressure ^(12)^.

According to the British Lymphology Society, BCRL causes the skin to expand, making it more vulnerable to injury, radiation-induced scarring, and skin infections. Patients with BCRL are educated on maintaining skin integrity and protection through an appropriate skin care regimen, including using low-pH soaps, creams, or lotions at appropriate times and for specific purposes, to prevent skin breakdown and bacterial colonisation ^(13)^.

Although numerous treatment options exist for BCRL, there is still no consensus on the most effective approach, highlighting the need for further studies exploring comprehensive therapy methods. A previous systematic review suggested effective lymphedema management may involve combined physical therapy ^(11)^. Therefore, we investigated the effect of a multimodal physical therapy approach in reducing UL lymphedema and increasing the shoulder range of motion (ROM) among women with BCRL.

**Methods**

**Subjects and study design**

This study was conducted in accordance with the Declaration of Helsinki, ensuring the ethical treatment of all participants involved. The institutional human ethics committee at the PSG Institute of Medical Sciences and Research granted ethical clearance for the study (PSG/IHEC/2023/Appr/Exp/173). Written informed consent was obtained from each patient before their inclusion in the study, allowing them to understand the nature of the research, the procedures involved, and any potential risks associated with the study.

A retrospective pre-post study was conducted using the data collected from women with BCRL treated at the oncology outpatient department from April 2021 to March 2022. They underwent screening by an oncologist, who referred them to physical therapy. A senior physiotherapist with a master's degree in Physical therapy and three years of experience in the oncology department treated them.

We obtained data based on the following criteria: Women aged above 18 who underwent tumor resection and at least two levels of axillary lymph node dissection with more than a 2 cm circumference difference between the two upper limbs (unilateral lymphedema stage 0-2) and had completed chemotherapy and radiotherapy were included in this study. Women with any history of neurological or musculoskeletal diseases or deformities in the ipsilateral UL affecting mobility, skin infection, current limb ischemia, venous thrombosis, edema due to impaired heart, kidney, and liver function, and current metastasis were excluded. The collected data at baseline assessment (commencement of the intervention) and at the end of the four weeks (end of Phase I) were used for statistical analysis.

**Sample size calculation:**

G*Power version 3.1.9.7 was used to determine the sample size. A paired t-test with an effect size of 0.8, an alpha error probability of 0.05, and a power (1-beta error probability) of 0.95 requires a minimum of 19 subjects.

**Intervention**

To ensure complete reporting of interventions, we used a template for intervention description and replication (TIDieR) checklist ^(14)^ (Table 1).

**Outcome measures**

The therapist measured the following variables at baseline and at the end of four weeks of intervention.

**Upper limb circumference measurement**

The circumference of the UL was measured using an inch tape measure at intervals of 4 cm up the arm from the ulnar styloid to the axilla (0 to 40 cm) and 4 and 8 cm below the hand from the ulnar styloid process. The sum of the circumferences in cm at each level was calculated for both limbs, and the difference between the limbs was taken for final analysis. Researchers have reported excellent intra- and inter-rater reliability when assessing lymphedema with a tape measure, with intraclass correlation coefficient scores ranging from 0.98 to 0.98 ^(15)^.

**Shoulder Range of Motion**

Shoulder flexion, extension, abduction, internal rotation, and external rotation in degrees were measured using a universal goniometer. Researchers have reported excellent test-retest reliability (ICC: 0.95 to 0.98) for assessing ROM using a goniometer  ^(16)^.

**Statistical analysis**

Descriptive statistics were computed for participant demographics. Paired t-tests were conducted to determine the significance of changes in UL circumference and shoulder ROM before and after the intervention. Cohen’s d was estimated for the effect of multimodal physical therapy on breast cancer-related lymphedema, categorising the effect as trivial (<0.2), small (0.2-0.5), medium (0.5-0.8), or high (>0.8). All statistical analyses were conducted using SPSS version 16.0 (SPSS Inc., IBM, Chicago, IL), with the risk of type I error set at P < 0.05.

**Results**

Table 2 provides an overview of the participants’ demographic characteristics. Of the 19 women diagnosed with BCRL, 18 had received radiation therapy. All of them had their lymph nodes dissected at level 3. The participants ranged in age from 44 to 74 years (mean ± SD, 59.57 ± 10.39 years). Most of the participants had stage 2A lymphedema. The mean height and weight of the participants were 151.6 ± 4.67 cm and 60.63 ± 8.63 kg, respectively, with a mean BMI of 26.36 ± 3.58 kg/m^2^.

Table 3 compares the outcomes from the baseline to the end of the four-week period. The intervention program significantly improved UL circumference and shoulder ROM (p < 0.05). Following the multimodal intervention, UL circumference was significantly reduced by 2.18 cm (95% CI: 1.54 cm, 2.68 cm; Cohen’s d = 1.35; p < 0.05), shoulder flexion increased by 46.31° (95% CI: -57.5°, -35.1°; Cohen’s d = 1.68; p < 0.05), shoulder extension increased by 10° (95% CI: -13.4°, -6.59°; Cohen’s d = 1.74; p < 0.05), shoulder abduction increased by 47.36° (95% CI: -60.35°, -34.38°; Cohen’s d = 1.67; p < 0.05), shoulder internal rotation increased by 18.94° (95% CI: -24.4°, -13.4°; Cohen’s d = 1.37; p < 0.05), and shoulder external rotation increased by 16.05° (95% CI: -20.03°, -12.07°; Cohen’s d = 1.51; p < 0.05).

Supplementary Table 1 presents the subgroup analysis of upper limb circumference and shoulder ROM across women with different lymphedema stages. These analyses demonstrated a progressive reduction in arm circumference and improvement in shoulder ROM after the intervention, with similar improvements observed irrespective of the lymphedema stage.

**Discussion**

This study aimed to reduce edema by decreasing interstitial fluid accumulation and improving lymphatic function through multimodal physical therapy. The literature has reported mixed results from various initiatives and treatment strategies proposed to address BCRL.

The current study found that women with BCRL had a significant reduction in their affected arm circumference (p < 0.05) with a larger effect size (Cohen's d = 1.35). Furthermore, Wang et al. discovered that the overall length of the arm circumference in the experimental group was less than that in the control group (126.39 cm vs. 145.26 cm), owing to the experimental group's participation in UL strengthening exercises and massage. They also said that restoring UL function necessitates lowering edema in the impacted limb, which then improves shoulder function.

Similarly, Borman et al. ^[17]^ conducted a study and found better improvements in the measurements of lymphedema (p <0.05) after 3 weeks of a combined protocol of complex decongestive therapy. This included skin care, manual lymphatic drainage, short-stretch multilayer bandaging, and lymphedema exercises ^[17]^. Moreover, Diab et al. ^[18]^ evaluated 30 women with BCRL and compared an intervention group that received IPC with Kinesio tape as an addition to complex decongestive therapy to a control group that underwent only complex decongestive therapy. Compared to the control group, the intervention group exhibited a substantial decrease in lymphedema size and a noticeable increase in shoulder ROM^[18]^.

Conversely, Blom et al. ^[9]^ stated that while compression garments combined with self-care initially produced a significant reduction in lymphedema relative volume at 6 months (p = 0.004) compared with self-care alone, this benefit was not sustained at 9 and 12 months. The compression garment group showed a 3.8% decrease in lymphedema volume after 6 months, while the non-compression garment group showed only a 0.1% increase (p < 0.001). Groups were similar at 9 and 12 months ^[9]^. These findings suggest that the effectiveness of compression garments may be limited to short-term management, highlighting the need for ongoing or adjunctive interventions to maintain long-term benefits ^[9]^.

In terms of improvements in shoulder ROM, flexion (46.31 ± 23.20; P <0.001) and abduction (47.36 ± 26.94; P <0.001) outperformed extension (10 ± 7.07; P <0.001) in the current study. These improvements reached the minimal detectable change (MDC) set by Rasmussen et al. ^[19]^ at 20.8^0^ and 10.2^0^ for abduction and flexion, respectively. Consistent with our findings, a previous review by Stuiver et al. ^[20]^ found that early shoulder exercises and MLD improved shoulder mobility for abduction and forward flexion in the initial weeks following breast cancer surgery. Early shoulder exercise improved forward flexion at one- and six-month follow-up, but not at 12 months ^[20]^. On contrast, a previous study by Otero et al. on 43 women with lymphedema found that IPC and complex physical therapy for first three weeks worked better together to improve shoulder flexion (5.6 ± 20.0; P = 0.04), extension (5.0 ± 9.5; P = 0.001), and abduction (11.4±30.7; P = 0.024) ^[21]^, but they did not meet the MDC set by Rasmussen et al. ^[19]^. Instead, Basha et al. ^[22]^ determined the effects of virtual reality and resistance exercise training on BCRL over eight weeks and found mean between-group differences of 13.0^0^, 21.17^0^, and 8.93^0^ for shoulder flexion, abduction, and external rotation. When compared to Rasmussen et al. ^[19]^, MDC values of 10.2^0^ for flexion and 20.8^0^ for abduction, the study by Basha et al. ^[22]^ showed improvements in flexion and abduction exceeded the MDC thresholds, indicating true functional changes beyond measurement error. The observed shift in external rotation did not exceed the MDC set by Rasmussen et al. ^[19]^, suggesting that clinically relevant improvements in this movement may not be possible. Similarly, Cho et al. ^[23]^ examined the effects of physical therapy combined with MLD for four weeks on shoulder function in 41 women with axillary web syndrome. They found that both groups significantly improved shoulder flexion and abduction, with both groups achieving a full range of motion (180°) and satisfying MDC requirements set by Rasmussen et al ^[22] [23]^.

In our study, improvements in internal rotation (18.94 ± 11.49; p = 0.000) exceeded those of external rotation (16.05 ± 8.26; p = 0.000) and had a larger effect size. However, Rasmussen et al. ^[19]^ specified that MDC values were only met for internal rotation (9.2) but not for external rotation (20.1) of the shoulder. Similarly, Luz et al. found that complex physical therapy (which includes regular exercises, skin care, manual lymphatic drainage, and multilayer bandage compression therapy) improved shoulder internal rotation (17.6 ± 32.6; p = 0.03) more than complex physical therapy combined with strength training.

Limitations, methodological considerations, and future recommendations are acknowledged. The retrospective pre–post design without a control group precludes causal inference, as observed improvements may partly reflect natural recovery, or concomitant therapies received outside the knowledge of the treating therapist. Furthermore, regression to the mean must be considered; patients exhibiting more severe lymphedema at baseline assessment may have experienced natural improvement over time, regardless of the multimodal intervention. Therefore, our results require careful interpretation, and future randomised or controlled clinical trials are required to validate these results.

The limited sample size (n = 19) constrains statistical power, increases susceptibility to individual variability, and raises the likelihood of Type II error. To address this, a sensitivity analysis was carried out using G*Power version 3.1.9.7 to determine the Minimum Detectable Effect (MDE) at 80% power and 5% significance. The MDE values (−0.54 cm for limb circumference, −3.20° for flexion, 1.31° for extension, −2.54° for abduction, 2.55° for internal rotation, and 1.56° for external rotation of the shoulder) suggest the study was adequately sensitive to detect clinically meaningful changes, as evident in Table 3, despite the small sample size. Even so, outcomes were only evaluated at baseline and four weeks, which prevented the assessment of long-term retention of effects. Future research with extended follow-up is needed to ascertain the durability of outcomes. The onset of lymphedema varied across participants, who were women aged 40 to 75 years and had undergone various types of breast surgery.

External validity is limited by the single-centre, retrospective design and the relatively brief follow-up period; however, internal validity remains sound due to the utilisation of objective assessment methods and standardised therapeutic protocols. The results are still clinically relevant for Indian women with BCRL, where limited resources and different levels of access to specialised rehabilitation services make pragmatic, multimodal physical therapy approaches even more important.

Finally, limb circumference was measured using a tape measure, a reliable method in the literature, reducing the possibility of bias. Nonetheless, measurements were carried out without blinding or duplicate evaluation, which may have resulted in measurement error. Patient-reported outcomes, such as overall quality of life and symptom load, were not measured, preventing a thorough assessment of the intervention's effectiveness. Future studies should therefore incorporate validated patient-reported measures alongside objective assessment.

**Conclusion**

The study findings indicate that our multimodal physical therapy approach effectively reduces excess arm volume and enhances shoulder ROM in BCRL, supporting its potential use in clinical practice for lymphedema management. However, randomised or controlled clinical trials are further required to confirm these findings.

**Declarations**

**Acknowledgements:** The authors greatly appreciate the guidance and encouragement provided by Dr. Balaji, the director of the PSG Institute of Oncology, Coimbatore.

**Conflict of interest:** The authors have not disclosed any conflicts of interest.

**Funding details:** No author has a financial stake in the results of this study.

**Data availability statement:** Data are available from the corresponding author upon reasonable request, subject to institutional review board approval.

**Authors’ contribution:** DV contributed to conceptualisation, data collection, analysis, and manuscript writing; MV contributed to data analysis, interpretation of results, and manuscript writing; and AA contributed to the data analysis, interpretation of results, critical review, and manuscript revision and editing. FM and NM contributed to critical review and manuscript editing. RKS and MV contributed to the data collection. All authors have read and approved the final version of the manuscript and agree with the order of presentation of the authors.

**References:**

1. Fitzmaurice C, Abate D, Abbasi N, Abbastabar H, Abd-Allah F, Abdel-Rahman O, et al. Global, regional, and national cancer incidence, mortality, years of life lost, years lived with disability, and disability-Adjusted life-years for 29 cancer groups, 1990 to 2017: A systematic analysis for the global burden of disease study. JAMA Oncol. 2019 Dec 1;5(12):1749–68.

2. Rupp J, Hadamitzky C, Henkenberens C, Christiansen H, Steinmann D, Bruns F. Frequency and risk factors for arm lymphedema after multimodal breast-conserving treatment of nodal positive breast Cancer - A long-term observation. Radiation Oncology. 2019 Mar 7;14(1).

3. Marchica P, Darpa S, Magno S, Rossi C, Forcina L, Capizzi V, et al. Integrated treatment of breast cancer-related lymphedema: A descriptive review of the state of the art. Vol. 41, Anticancer Research. International Institute of Anticancer Research; 2021. p. 3233–46.

4. Tandra P, Kallam A, Krishnamurthy J. Identification and Management of Lymphedema in Patients With Breast Cancer [Internet]. Vol. 15, J Oncol Pract. 2019. Available from: https://doi.org/10.

5. Donahue PMC, MacKenzie A, Filipovic A, Koelmeyer L. Advances in the prevention and treatment of breast cancer-related lymphedema. Vol. 200, Breast Cancer Research and Treatment. Springer; 2023. p. 1–14.

6. Davies CC, Levenhagen K, Ryans K, Perdomo M, Gilchrist L. An Executive Summary of the APTA Academy for Oncologic Physical Therapy Clinical Practice Guideline: Interventions for Breast Cancer-Related Lymphedema. Vol. 38, Rehabilitation Oncology. Lippincott Williams and Wilkins; 2020. p. 103–9.

7. Hemmati M, Rojhani-Shirazi Z, Zakeri ZS, Akrami M, Salehi Dehno N. The effect of the combined use of complex decongestive therapy with electrotherapy modalities for the treatment of breast cancer-related lymphedema: a randomized clinical trial. BMC Musculoskelet Disord. 2022 Dec 1;23(1).

8. Atef D, Elkeblawy MM, El-Sebaie A, Abouelnaga WAI. A quasi-randomized clinical trial: virtual reality versus proprioceptive neuromuscular facilitation for postmastectomy lymphedema. J Egypt Natl Canc Inst. 2020 Dec 1;32(1).

9. Blom KY, Johansson KI, Nilsson-Wikmar LB, Brogårdh CB. Early intervention with compression garments prevents progression in mild breast cancer-related arm lymphedema: a randomized controlled trial. Acta Oncol (Madr). 2022;61(7):897–905.

10. Hettrick H, Ehmann S, McKeown B, Bender D, Blebea J. Selecting appropriate compression for lymphedema patients: American Vein and Lymphatic Society position statement. Phlebology. 2023 Mar 1;38(2):115–8.

11. Ezzo J, Manheimer E, Mcneely ML, Howell DM, Weiss R, Johansson KI, et al. Manual lymphatic drainage for lymphedema following breast cancer treatment. Vol. 2015, Cochrane Database of Systematic Reviews. John Wiley and Sons Ltd; 2015.

12. Venugopalan M, Divya Varshini R, Veyilmuthu R, Bhuvaneswari CKV. Exercise benefits following breast cancer surgery – a narrative review. Fizjoterapia Polska. 2024;2024(4):392–5.

13. Anita Hobday. Skin Care for people with Lymphoedema. Br J Community Nurs [Internet]. 2023;28:S12–4. Available from: www.thebls.com

14. Hossain M. A Study on Behavioral and Biological Risk Factors Determination of Non-Communicable Diseases at Narayanganj. 2017.

15. Liang M, Chen Q, Peng K, Deng L, He L, Hou Y, et al. Manual lymphatic drainage for lymphedema in patients after breast cancer surgery: A systematic review and meta-analysis of randomized controlled trials. Medicine. 2020 Dec 4;99(49).

16. Mullaney MJ, McHugh MP, Johnson CP, Tyler TF. Reliability of shoulder range of motion comparing a goniometer to a digital level. Physiother Theory Pract. 2010 Jul;26(5):327

17. Borman P, Yaman A, Yasrebi S, Pınar İnanlı A, Arıkan Dönmez A. Combined Complete Decongestive Therapy Reduces Volume and Improves Quality of Life and Functional Status in Patients with Breast Cancer-Related Lymphedema. Clinical Breast Cancer. 2021 Aug;22(3).

18. Diab HA, Asham HN, Aboelnour NH, Alagizy HA. Effect of Intermittent Pneumatic Compression in Combination with Kinesiotape on Post Mastectomy Lymphedema. The Egyptian Journal of Hospital Medicine. 2021 Oct 1;85(1):2794–9.

19. Rasmussen GHF, Kristiansen M, Arroyo-Morales M, Voigt M, Madeleine P. Absolute and relative reliability of pain sensitivity and functional outcomes of the affected shoulder among women with pain after breast cancer treatment. Calvo-Lobo C, editor. PLOS ONE. 2020 Jun 3;15(6):e0234118.

20. Stuiver MM, ten Tusscher MR, Agasi-Idenburg CS, Lucas C, Aaronson NK, Bossuyt PM. Conservative interventions for preventing clinically detectable upper-limb lymphoedema in patients who are at risk of developing lymphoedema after breast cancer therapy. Cochrane Database of Systematic Reviews. 2015 Feb 13;

21. Pajero Otero V, García Delgado E, Martín Cortijo C, Rodríguez Ramos ML, De Carlos Iriarte E, Gil García A, et al. Intensive complex physical therapy combined with intermittent pneumatic compression versus Kinesio taping for treating breast cancer‐related lymphedema of the upper limb: A randomised cross‐over clinical trial. European Journal of Cancer Care. 2022 May 31;31(5).

22. Basha MA, Aboelnour NH, Alsharidah AS, Kamel FH. Effect of exercise mode on physical function and quality of life in breast cancer–related lymphedema: a randomized trial. Supportive Care in Cancer. 2021 Oct 20;30(3):2101–10.

23. Cho Y, Do J, Jung S, Kwon O, Jeon JY. Effects of a physical therapy program combined with manual lymphatic drainage on shoulder function, quality of life, lymphedema incidence, and pain in breast cancer patients with axillary web syndrome following axillary dissection. Supportive Care in Cancer. 2015 Nov 5;24(5):2047–57.

‌

**Table1. Template for intervention description and replication (TIDieR) checklist.**

| **TiDieR Items** | **Description** |
| --- | --- |
| The name of the intervention | A multimodal physical therapy approach comprising MLD, IPC, compression garments, exercises (along with compression garments), and meticulous skin care. |
| Rationale | - MLD stimulates lymph flow, generates new lymphatic channels, and softens fibrotic tissue. - IPC forces tissue fluid to areas where lymphatic outflow is typical, restoring the function of the disrupted lymphatics to normal. - Compression garments improve drainage by utilizing the working pressures generated by muscular activity. - Exercises bring a "muscle pump" by generating muscular contractions, which improve lymph flow and protein absorption. Additionally, the earliest lymphatic capillaries expand and widen in response to changes in tissue pressure, which facilitates the easier entry of interstitial fluid into the lymphatic system. - Meticulous skin care improves skin integrity and prevents new infections. |
| Materials used in the intervention | An IPC device model AIROS 6 (AIROS Medical Inc. 2501 Monroe Blvd., Suite 1200, Audubon, United States- 19403), class II short-stretch home-made customized compression garments, ball, towel, chair, cot, pillow, and wall |
| Intervention procedures | The subjects removed clothing and jewelry from the treatment area before treatment, and the therapist did a skin inspection to rule out infections or wounds.  **MLD:** Subjects sat in a comfortable position and performed abdominal breathing. Using three fingers, the therapist gently stretched the skin over the lymph nodes above the clavicle on both sides. Using the flat surface of the fingers, the therapist gave a gentle pressure over both armpits in a circular motion to pump the lymph nodes. The therapist massaged using the flat surface of the hand in a circular motion: the anterior chest, the involved armpit to (across) the uninvolved armpit, the front, back, and outside of the upper arm (moved up from the elbow to the shoulder); the anterior and posterior aspects of the elbow; the forearm (from the wrist to the elbow). The therapist used their hands to massage all sides of the wrist in a half-circular motion. Rub fingers from knuckle to wrist, palms from center to edge, and nail buds to palm. Followed by abdominal breathing.  **IPC:** Subjects were asked to wear their own stockinette. A multichambered garment of the AIROS 6 equipment was applied to the patient’s upper limb ^[^.  **Compression garments:** Subjects were asked to wear Class II short-stretch home-made customized compression garments during the day.  **Exercises:** Subjects engaged in hand ball squeezes, wrist and elbow movements, shoulder shrugging, wand exercise, wall climbing, towel exercise, shoulder bracing, butterfly stretch, overhead clasped hands stretch, shoulder rotations (both clockwise and anticlockwise), and upper body pushups.  **Skin care:** The therapist educated subjects about meticulous skin care, which includes skin inspection, skin hygiene, and the prevention of skin injury. They had to check the skin every day for redness, scratches, abrasions, or wounds. Daily skin care routines should focus on gently cleansing the skin with a mild cleanser free of soap (as soaps can change the skin's natural pH and remove the protective sebum layer), patting dry the skin with a gentle motion, and moisturizing the skin with an emollient to replenish lost sebum and retain moisture. They were asked to follow these measures to prevent skin injury: application of sunscreen, usage of insect repellent to prevent bites, usage of electric razors in preference to blades, wearing gloves during gardening, avoiding injections and blood samples on the affected side, avoiding hot pack application, avoiding tight-fitting clothes, watch bands, bracelets, rings, and bra straps. |
| Provider | The therapist had provided all the procedures to the subjects. |
| Mode of intervention delivery | The program includes individual therapy sessions and a home-based exercise program. |
| Setting of the intervention | Assessments and interventions were conducted at the Department of Oncology, PSG Hospitals. |
| Dosage | The therapist provided all interventions to the subjects for four weeks (five days per week).  **MLD:** 30 minutes (five days per week).  **IPC:** The Lymphoedema Framework recommends using IPC compression levels between 30 and 60 mmHg, adjusting them based on therapy response, and using them as tolerated. Pneumatic compression pressure for UL treatment should be 40 mm Hg, 50 mm Hg, or 30 mm Hg; these values correlate to the 30-60 mm Hg recommended by ISL. Similar to that, throughout four weeks, five days a week, a pressure of 40 to 60 mmHg was applied for 30 minutes at a time.  **Compression garments:** pressure of 25–35 mmHg during the day (everyday).  **Exercises:** During therapy sessions, subjects performed one set of exercises, 15 repetitions each, along with a compression garment. At home, they did four sets of exercises, 15 repetitions each wearing a compression garment. |
| Tailoring | Subjects experiencing fatigue during exercise took rest intervals between the exercise programs. |
| Modifications | Not applicable |
| Fidelity assessment | All the participants were asked to mark their home exercise routine in their logbook, and it was counterchecked by the therapist when the participants had come for therapy sessions. Adherence to other treatment modalities was directly monitored by the therapist, and exercises were performed under supervision during therapy sessions. |

IPC: Intermittent pneumatic compression; MLD: Manual lymphatic drainage

**Table 2. Demographic characteristics (n=19)**

| **S. No** | **Characteristics** | **Values** |
| --- | --- | --- |
| 1. | **Age (years)** | 59.57 (10.39) * |
| 2. | **Side**  Right  Left | 6 (32%) ^$^  13 (68%) ^$^ |
| 3. | **Lymphedema stage**  1  2A  2B | 6 (32%) ^$^  8 (42%) ^$^  5(26%) ^$^ |
| 4. | Height (cm)  Weight (kg)  Body mass Index (kg/m^2^)  Normal  Overweight  Obese | 151.6 (4.67) *  60.63 (8.63) *  26.36 (3.58) *  7 (37%) ^$^  9 (47%) ^$^  3 (16%) ^$^ |

*Mean (standard deviation); ^$^n (%)

**Table 3. Comparison of outcomes before and after intervention among women with BCRL**

| **S.no** | **Variables** | **Baseline**  **Mean (SD)** | **After four weeks**  **Mean (SD)** | **Mean difference**  **(post-intervention – pre-intervention scores)** | **95% CI** | **Cohen’s d** | **p value** |
| --- | --- | --- | --- | --- | --- | --- | --- |
| 1. | **Upper-limb circumference (cm)** | 4.01 (1.87) | 1.90 (1.17) | 2.11 | 1.54, 2.68 | 1.35 | **p<0.001** |
| 2. | **Shoulder ROM (degrees)**  Flexion | 105.26 (29.49) | 151.5(25.38) | 46.31 | -57.5, -35.1 | 1.68 |  |
|  | Extension | 25.7 (4.79) | 35.7 (6.51) | 10.00 | -13.4, -6.59 | 1.74 |  |
|  | Abduction | 94.21 (29.87) | 141.58 (26.61) | 47.36 | -60.35, -34.38 | 1.67 |  |
|  | Internal rotation | 38.42 (12.02) | 57.36 (15.30) | 18.94 | -24.4, -13.4 | 1.37 |  |
|  | External rotation | 29.47 (9.53) | 45.52 (11.53) | 16.05 | -20.03, -12.07 | 1.51 |  |

ROM: Range of motion; SD: Standard deviation; 95% CI: 95% Confidence interval; p value: obtained with paired “t” test

Note: The mean difference (MD) and 95% confidence intervals (CI) are determined as post-intervention minus baseline. Negative values imply improvement (higher post-intervention scores than baseline), whereas positive ones suggest a decrease.
