# Supplementary material for: Effects of a multimodal physical therapy approach on breast cancer-related lymphedema: a retrospective pre-post study
Source: Sci Rep. 2025 Nov 21;15:41175. doi: 10.1038/s41598-025-27786-0 (PMC12638769; doi:10.1038/s41598-025-27786-0)
Supplement: Supplementary file 2 — Supplementary Material 2 [file 41598_2025_27786_MOESM2_ESM.docx]

**Supplementary Table 1. Subgroup analysis of lymphedema stages in relation to upper limb circumference (cm) and shoulder range of motion (degrees)**

| **Stages of lymphedema** | **Variables** | **Baseline**  **Mean (SD)** | **After four weeks**  **Mean (SD)** | **Mean difference (post-intervention – pre-intervention scores)** | **95% CI** |
| --- | --- | --- | --- | --- | --- |
| **Lymphedema stage 1 (n=6)** | Upper Limb Circumference | 3.2 (1.03) | 1.12 (0.68) | 2.08 | 1.35, 2.79 |
|  | Shoulder flexion | 138.32 (16.02) | 173.32 (4.08) | 35 | -48.27, -21.72 |
|  | Shoulder extension | 29.16 (4.91) | 40 (5.47) | 10.84 | -18.55, -3.10 |
|  | Shoulder abduction | 126.6 (20.65) | 165 (8.36) | 39 | -53.05, -23.61 |
|  | Shoulder internal rotation | 49.16 (10.20) | 74.16 (5.84) | 25 | -37.85, -12.14 |
|  | Shoulder external rotation | 36.66 (10.32) | 57.5 (6.12) | 20.84 | -30.46, -11.20 |
| **Lymphedema stage 2A (n=8)** | Upper Limb Circumference | 3.40 (1.51) | 1.73 (1.07) | 1.67 | 1.04, 2.28 |
|  | Shoulder flexion | 96.25 (21.99) | 141.25 (30.90) | 45 | -66.43, -23.56 |
|  | Shoulder extension | 25 (3.77) | 34.37 (7.76) | 9.37 | -16.93, -1.81 |
|  | Shoulder abduction | 86.25 (18.46) | 131.25 (31.02) | 45 | -70.76, -19.23 |
|  | Shoulder internal rotation | 35 (10.69) | 46.87 (12.51) | 11.87 | -20.51, -3.23 |
|  | Shoulder external rotation | 27.5 (8.45) | 38.12 (10.32) | 10.62 | -16.29, -4.95 |
| **Lymphedema stage 2B (n=5)** | Upper Limb Circumference | 5.97 (1.96) | 3.09 (0.95) | 2.07 | 0.62, 5.14 |
|  | Shoulder flexion | 80 (14.14) | 142 (13.03) | 62 | -91.64, -32.35 |
|  | Shoulder extension | 24 (5.47) | 33 (2.73) | 9 | -17.04, -0.90 |
|  | Shoulder abduction | 68 (17.88) | 130 (14.57) | 62 | -100.42, -23.57 |
|  | Shoulder internal rotation | 31 (7.41) | 43 (5.70) | 12 | -23.27, -0.72 |
|  | Shoulder external rotation | 24 (5.47) | 43 (5.70) | 19 | -24.19, -13.80 |

SD: Standard deviation; 95% CI: 95% Confidence interval; p value: obtained with paired “t” test

Note: The mean difference and 95% confidence intervals (CI) are determined as post-intervention minus baseline. Negative values imply improvement (higher post-intervention scores than baseline), whereas positive ones suggest decrease.
